# Development of a Cleaved Probe-Based Loop-Mediated Isothermal Amplification Assay for Rapid Detection of African Swine Fever Virus

**DOI:** 10.3389/fcimb.2022.884430

**Published:** 2022-05-26

**Authors:** Songqi Wang, Haiyan Shen, Qijie Lin, Jun Huang, Chunhong Zhang, Zhicheng Liu, Minhua Sun, Jianfeng Zhang, Ming Liao, Yugu Li, Jianmin Zhang

**Affiliations:** ^1^Key Laboratory of Zoonoses Prevention and Control of Guangdong Province; The Research Center for African Swine Fever Prevention and Control; College of Veterinary Medicine, South China Agricultural University, Guangzhou, China; ^2^Maoming Branch Center of Guangdong Laboratory for LingNan Modern Agricultural Science and Technology; Key Laboratory of Livestock Disease Prevention of Guangdong Province, Scientific Observation and Experiment Station of Veterinary Drugs and Diagnostic Techniques of Guangdong Province, Ministry of Agriculture and Rural Affairs, Institute of Animal Health, Guangdong Academy of Agricultural Sciences, Foshan, China; ^3^College of Life Science and Engineering, Foshan University, Guangzhou, China

**Keywords:** African swine fever virus, rapid detection, RNase H2, CP-LAMP, point-of-care testing, smartphone quantitation

## Abstract

African Swine Fever (ASF), caused by African swine fever virus (ASFV), is a highly contagious and lethal viral disease of pigs. However, commercial vaccines are not yet available, and neither are drugs to prevent or control ASF. Therefore, rapid, accurate on-site diagnosis is urgently needed for detection during the early stages of ASFV infection. Herein, a cleaved probe-based loop-mediated isothermal amplification (CP-LAMP) detection method was established. Based on the original primer sets, we targeted the ASFV 9GL gene sequence to design a probe harboring a ribonucleotide insertion. Ribonuclease H2 (RNase H2) enzyme activity can only be activated when the probe is perfectly complementary, resulting in hydrolytic release of a quencher moiety, and consequent signal amplification. The method displayed robust sensitivity, with copy number detection as low as 13 copies/µL within 40 min at constant temperature (62°C). Visualization of the fluorescence product was employed using a self-designed 3D-printed visualization function cassette, and the CP-LAMP method achieved specific identification and visual detection of ASFV. Moreover, coupling the dual function cassette and smartphone quantitation makes the CP-LAMP assay first user-friendly, cost-effective, portable, rapid, and accurate point-of-care testing (POCT) platform for ASFV.

## Introduction

African swine fever (ASF), an acute, hemorrhagic, virulent disease caused by ASV virus (ASFV), affects domestic pigs and wild boar (Normile, 2018; Ma et al., 2020). ASFV infection is characterized by rapid onset and a mortality rate near 100% for the most acute infections, depending on the viral strain (Galindo and Alonso, 2017). ASF has a devastating economic impact on the global pig industry (He et al., 2020; Yoon et al., 2020). Currently, there are no effective drugs or commercial vaccines to prevent and/or control ASF. Hence, a rapid on-site method is urgently needed for ASFV detection during the early stages of ASFV infection.

The World Organization for Animal Health currently recommends viral isolation, antigen detection, and molecular diagnostic methods for ASFV (Sánchez-Vizcaíno and Mur, 2013). However, viral isolation is laborious and ill-suited to in-field practices, while the presence of antibodies can disrupt antigen detection (Cadenas-Fernández et al., 2019). For ASFV detection, conventional and real-time PCR methods are considered the most reliable (King et al., 2003; Fernández-Pinero et al., 2013), but they are not sufficiently sensitive, and they cannot achieve quantitative analysis of small quantities of virus DNA during amplification. Real-time fluorescence quantitative PCR (qPCR) has been applied for testing ASFV, which can achieve quantitative analysis at low concentrations of samples using standard sample templates. However, these two methods required laboratory-based equipment and advanced expertise.

Loop-mediated isothermal amplification (LAMP) is a highly specific, simple, sensitive, and rapid technique for pathogen detection (Tomita et al., 2008). In LAMP assays, the target DNA or RNA template is amplified under isothermal conditions (60−65°C) using a DNA polymerase possessing strong strand displacement activity, and a set of primers that recognizes six distinct genomic regions. LAMP does not require expensive equipment, making it potentially ideal for clinical field testing. In addition, The LAMP assay results can be visualized with intercalating dyes such as ethidium bromide and SYBR Green (Tomita et al., 2008), or complexometric calcein (Hill et al., 2008) and hydroxyl naphthol blue (HNB) (Goto et al., 2009), a fluorescent metal indicator, under a UV lamp. However, distinguishing weak color changes from negative reactions is challenging, increasing the risk of inaccurately interpreting the results, especially at low sample concentrations. LAMP results can be judged quantitatively using real-time thermocyclers that detect fluorescence, or a real-time turbidimeter (Hill et al., 2008). However, LAMP cannot achieve quantification through analysis of color changes of fluorescent intercalating dyes.

To overcome these limitations, we aimed to develop a rapid, sensitive, cleaved probe-based LAMP (CP-LAMP) detection method. In this method based on traditional LAMP, we replaced one primer with a loop primer probe containing a ribonucleotide insertion; only when the base sequence perfectly matches the DNA-RNA mutant target can it be cleaved by the enzyme RNase H2. When the reporter fluorophore and the quencher fluorophore of the loop primer probe are separated, the fluorescent signal is generated, which can be read by a real-time thermocycler. Our method can detect ASFV quantitatively based on a standard curve, and it does not require addition of separate dyes, which effectively avoids contamination and false-positive results. With the help of a portable self-designed 3D-printed visualization function cassette to read the results, detection of ASFV is made more convenient for clinical diagnosis, and it meets the requirements of POCT.

## Materials and Methods

### Primer Design

Sequence data from publicly accessible databases were used to generate consensus sequences by aligning the genomes of ASFV isolates. Primers were designed based on the 9GL gene sequence of ASFV isolate Pig/HLJ/2018 (GenBank: MK333180.1), which is highly conserved, and targeted for specific primer design using Primer Explorer V5. All oligonucleotide primers and probes used in this research were synthesized by Sangon Biotech (Shanghai) Co., Ltd. (Shanghai, China).

### Standard Plasmid

The 9GL gene was chemically synthesized by Sangon Biotech (Shanghai) Co., Ltd., and inserted into the pUC57 plasmid (herein referred to as pUC57-9GL). The resulting construct was transformed into *Escherichia coli* DH5α cells, then extracted using an Endo-free Plasmid Mini Kit (D6950, Omega Bio-tek, USA) according to the manufacturer’s instructions. Plasmid concentrations were measured by spectrophotometry, converted to copy numbers, and plasmid were used as templates for sensitivity assays.

### CP-LAMP Reaction Conditions

In-tube CP-LAMP reactions consisted of a 25 μL reaction mixture containing 8 U of Bst 2.0 DNA polymerase, 100 µmol MgSO_4_, 2.5 µL of 10× buffer (New England Biolabs Inc.), 0.1 U/µL RNase H2 Enzyme (catalog no. 11-02-12-01, Integrated DNA Technologies), 4 µL of dNTPs (TransGenBiotech), and 2.5 µL of DNA sample. Mineral oil was applied to the surface to prevent contamination before lid closure. The reaction procedure was performed at 1 cycle/min for 60 cycles using a CFX96 Touch Real-time PCR Detection System (Bio-Rad). The CP-LAMP assay could be completed in less than 40 min at 62°C, and the reaction was monitored using a real-time PCR instrument.

### Specificity of CP-LAMP

Genomic DNA from African swine fever virus (ASFV), porcine circovirus type 2 (PCV2), pseudorabies virus (PRV), and porcine parvovirus (PPV) was used to evaluate the specificity of the CP-LAMP method. Total RNA from classic swine fever virus (CSFV), transmissible gastroenteritis virus (TGEV), porcine reproductive respiratory syndrome virus (PRRSV), and porcine epidemic diarrhea virus (PEDV) was extracted and reverse-transcribed into cDNA for use in specific testing.

### Sensitivity of CP-LAMP

To evaluate the limit-of-detection (LOD) of the ASFV CP-LAMP assay, a 10-fold dilution series of pUC57-9GL in deionized water ranging from 1.3×10^6^ to 1.3 copies/µL was prepared. Each solution was tested in triplicate in a 96-well PCR plate and heated at 62°C for 40 min. Finally, experimental data were used to establish a standard curve.

### Stability and Repeatability of CP-LAMP

To measure the repeatability of this method, standard solutions of plasmid pUC57-9GL in the range of 1.3×10^6^ to 1.3×10^2^ copies/µL were used as templates. Under the same conditions, CP-LAMP amplification was performed and each dilution tested in triplicate. Finally, the coefficient of variation (CV) was calculated according to its cycle threshold (Ct) value to evaluate the repeatability of the measurement.

### Comparison of CP-LAMP With Conventional PCR (For Diagnostic Sensitivity)

Diagnostic performance was evaluated by testing 61 DNA samples provided by the Research Center for African Swine Fever Prevention and Control, South China Agricultural University, Guangzhou, People’s Republic of China. The CP-LAMP assay performance was directly compared with the conventional PCR amplification and TaqMan probe real-time PCR of ASFV (King et al., 2003; McKillen et al., 2010).

For the PCR assay, we performed as the Chinese national standard: Diagnostic techniques for African Swine Fever (GB/T 18648-2020). The primers were designed to target the conserved region of ASFV B646L gene, F-PPA-1: 5’-AGTTATGGGAAACCCGACCC-3’, R-PPA-2: 5’-CCCTGAATCGGAGCATCCT-3’, the primer concentration was 10μM, and the amplification band was 257 bp. The conventional PCR was performed in a 20μL reaction volume containing 10 μL Premix Taq, 1 μL each primer, 6 μL H_2_O and 2 μL of the genomic DNA. The tube was sealed, and centrifuged briefly. Positive, negative, and blank controls were included for each time of the conventional PCR performed. Thermal cycling involved a 95°C pre-denaturation step for 10 min, followed by 40 cycles of denaturation at 95°C for 15 s, annealing at 62°C for 30 s, extension at 72°C for 30 s, and a final extension at 72°C for 7 min. For PCR amplification product electrophoresis, 5 µL of 6 × loading buffer was added to each PCR product, mixed, and 8 µL was run on a 2% agarose gel prepared with 1 × TAE buffer, and electrophoresed for 30−40 min. After electrophoresis, the agarose gel was placed in a gel imager to observe the results.

### Assembly of the 3D-Printed Visualization Function Cassette for Detection

We prepared a hand-held portable cassette device box 122 mm long, 82 mm wide, and 70 mm deep in previous work (Wen et al., 2021). The box is powered by rechargeable portable lithium batteries, which are connected in series to eight light-emitting diodes (LEDs) with an emission peak of 495 nm. When energized, the light source passes through a 495 nm band-pass filter between the LED lamp and the centrifuge tube, and illuminates the reaction solution at 495 nm, causing the fluorescent group to emit light. In order to facilitate observation and eliminate the overlap of excitation light and emission light, a plane reflector with a 45°C angle was placed on the opposite side of the tube body, and the reaction result graph can be obtained using a mobile phone.

## Results

### Primer Design, Optimization, and establishment of the Basic Reaction System

**Figure 1:**
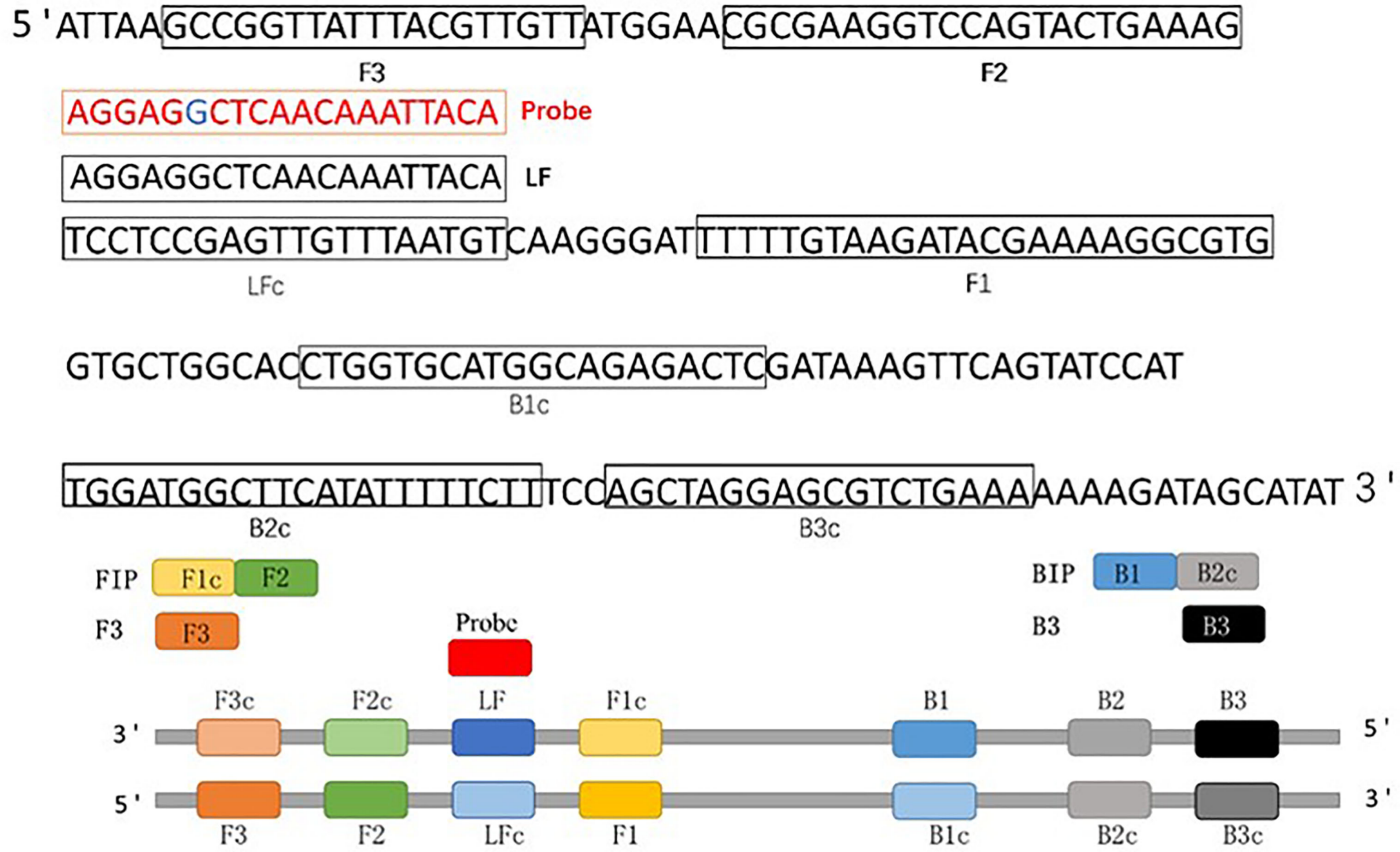
Schematic diagram of the 9GL gene showing the position and composition of ASFV CP-LAMP primers and probe. F3, forward outer primer (F3); B3, backward outer primer; FIP (F1c+F2), forward inner primer; BIP (B1+B2c), backward inner primer; LF, loop forward primer; Probe, cleaved probe.

Optimal CP-LAMP primers were selected by aligning the sequences of ASFV 9GL genes from the from NCBI database to identify the highly-conserved region for primer design. Five sets of candidate primers were selected and synthesized (**Table 1**). Moreover, we targeted this fragment of the ASFV 9GL gene to design a new reporter dye and a quencher-modified allelic discrimination cleaved probe with a ribonucleotide insertion (**Figure 1** and **Table 1**). Upon perfect matching with the ribonucleotide mutant site, the RNase H2 hydrolytic mechanism is activated, and release of the quencher generates an amplified signal. Conversely, a signal is not generated with mismatching ribonucleotides. Thus, robust specific detection of the ASFV 9GL gene was achieved.

**Table 1:** Primers and probe information of the CP-LAMP assay.

Name	Sequence (5’→3’)
F3	GCCGGTTATTTACGTTGTT
B3	TTTCAGACGCTCCTAGCT
FIP	CACGCCTTTTCGTATCTTACAAAAACGAAGGTCCAGTACTGAAAG
BIP	CTGGTGCATGGCAGAGACTCAAGAAAAATATGAAGCCATCCA
LF	ACATTAAACAACTCGGAGGA
probe	FAM-ACATTAAACAACTC ***G*** (RNA)GAGGA-BHQ1

The bold and underline letter indicates that the base is a ribonucleotide.

**Figure 2:**
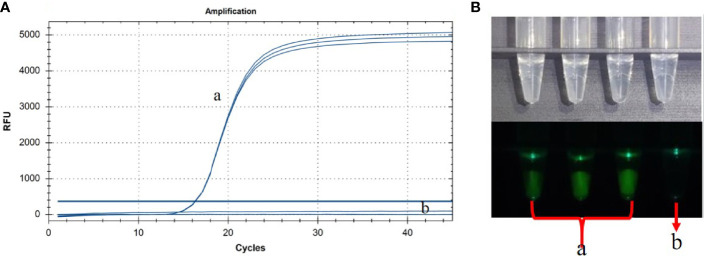
Basic reaction system was performed in real-time PCR instrument **(A)** and the results were observed using 3D-printed visualization function cassette **(B)**. (a) ASFV standard plasmid (in triplicate test). (b) Negative control.

Based on our previous report (Shen et al., 2020), we successfully set up a basic reaction system using standard plasmids. The reaction system contained 2.5 μL of buffer (10×), 1.5 μL of MgSO_4_, 4 μL of dNTPs, 8 U/µL of Bst 2.0 WarmStart DNA polymerase (1 µL), 0.1 U/µL of RNase H2 Enzyme (0.3 µL), 0.3 µL of probe (10 µM), 4 µL of FIP/BIP primer (10 µM), 0.5 µL of F3/B3 primer (10 µM), 1.5 µL of loop primer (10 µM), and 2.5 µL of DNA sample. The mixture was made up to 25 µL with deionized water. The ASFV standard plasmid was successfully detected with good repeatability and reaction efficiency (**Figure 2A**). In order to facilitate the clinical testing, we use a self-designed 3D-printed visualization function cassette to establish the point-of-care testing (POCT) platform for ASFV. After the CP-LAMP reaction, the tubes containing the reaction mixtures were placed in the cassette and the results can be visualized using a smartphone (**Figure 2B**).

**Figure 3:**
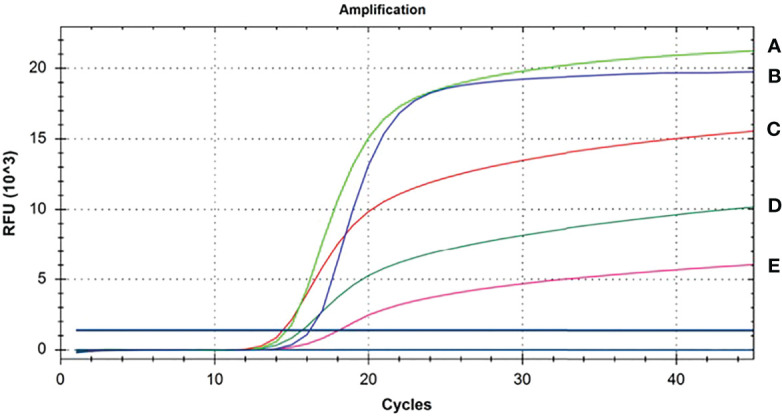
Temperature optimization results. **(A)** 61°CC. **(B)** 60°CC, **(C)** 62°CC, **(D)** 64°CC, **(E)** 63°CC.

To optimize the reaction temperature of the CP-LAMP assay, reaction mixtures were tested from 60 to 64°C at 1°C intervals for 60 min. There was optimal reaction efficiency and amplification efficiency at 62°C, hence this temperature was chosen for subsequent testing (**Figure 3**).

To confirm the optimal reaction duration of the CP-LAMP assay, fluorescence values were compared and analyzed throughout the entire reaction, and values peaked then tended to remain constant after 40 min. Based on amplification efficiency and total detection time, a 40 min reaction duration was considered most appropriate.

### Specificity of Test Results

**Figure 4:**
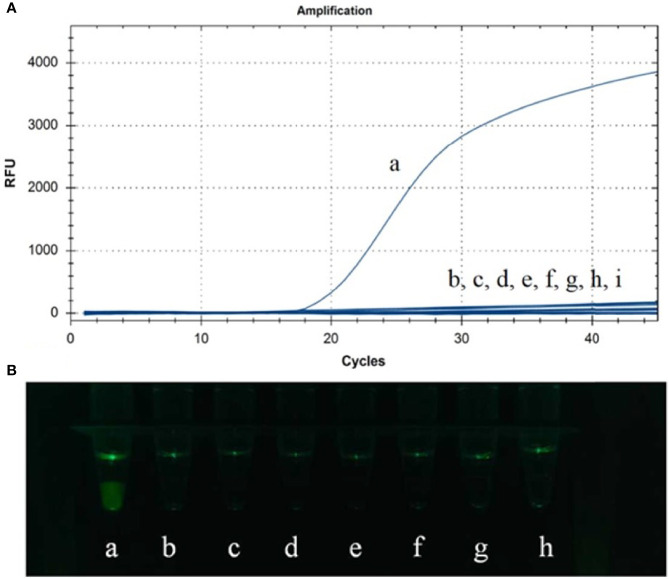
Specificity analysis was performed in real-time PCR instrument **(A)** and observed by the 3D-printed visualization function cassette **(B)**. (a) ASFV. (b) PRV. (c) PCV2. (d) CSFV. (e) PRRSV. (f) PPV. (g) PEDV. (h) TGEV. (i) Negative control.

Genomic DNAs or cDNAs of ASFV, PRV, PCV2, PPV, CSFV, PRRSV TGEV and PEDV were determined by CP-LAMP to evaluate the specificity. As shown in **Table 2** and **Figure 4**, reaction systems containing genomic DNA of ASFV gave excellent signal in the assay, while reaction systems containing DNAs or cDNAs from the other seven pathogens did not generate detectable signals. Therefore, the CP-LAMP assay displayed good specificity, and only amplified the 9GL gene DNA of ASFV.

**Table 2:** Specificity of the CP-LAMP assay.

Pathogen	ASFV	PRV	PCV2	PPV	CSFV	PRRSV	PEDV	TGEV
Result	+	–	–	–	–	–	–	–

### Detection Limit of Test Results

**Figure 5:**
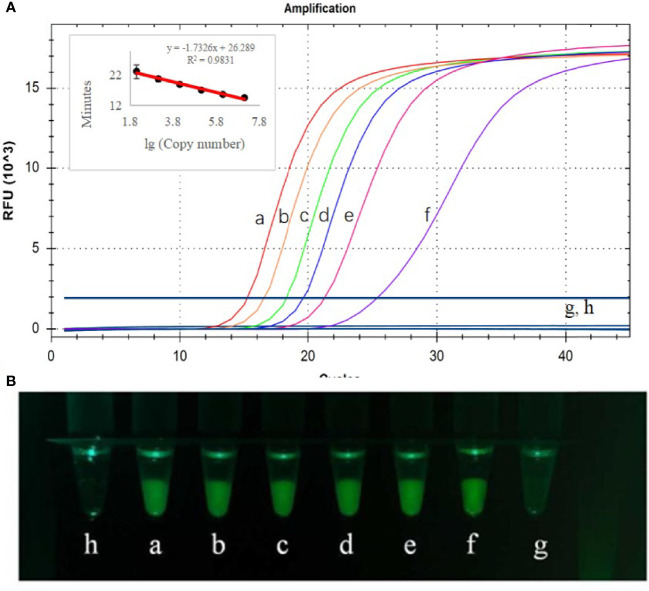
Detection limit analysis used the real-time PCR instrument **(A)** and observed by the 3D-printed visualization function cassette **(B)**. CP-LAMP was used tested using ASFV plasmid diluted to various concentrations. (a) 1.3×10^6^ copies/µL. (b) 1.3×10^5^ copies/µL. (c) 1.3×10^4^ copies/µL. (d) 1.3×10^3^ copies/µL. (e) 1.3×10^2^ copies/µL. (f) 1.3×10^1^ copies/µL. (g) 1.3×10^0^ copies/µL. (h) Negative control.

Sensitivity testing of CP-LAMP was performed by using the real-time PCR instrument, and the results showed that plasmid concentrations from 1.3×10^6^ copies/µL to 1.3 copies/µL were amplified successfully (**Figure 5**). Therefore, the detection limit was 13 copies/µL, analysis could be completed within 40 min, the standard curve equation was y = -1.7326x + 26.289 (R^2^ = 0.9831), and there was an excellent correlation between copy number the reaction duration. Moreover, the results observed by the 3D-printed visualization function cassette are consistent with the results observed by real-time PCR instrument. In all, these results demonstrated that the CP-LAMP assay established in this study to detect ASFV was a sensitive probe-based real-time LAMP method.

### Repeatability of Test Results

In the same laboratory, using the same instrument, each sample was replicated three times over a short period of time. The CV values of three repeated experiments were all less than 0.05 (**Table 3**), indicating that the method had good reproducibility.

**Table 3:** Reproducibility of the CP-LAMP method.

Plasmid concentration(copies/μL)	Intra-assay coefficient of variation	Inter-assay coefficientof variation
1.3×10^6^	3.01%	1.98%
1.3×10^5^	1.96%	0.11%
1.3×10^4^	4.20%	0.83%
1.3×10^3^	3.41%	1.87%
1.3×10^2^	4.54%	0.58%

### Application of CP-LAMP to Clinical Samples

**Figure 6:**
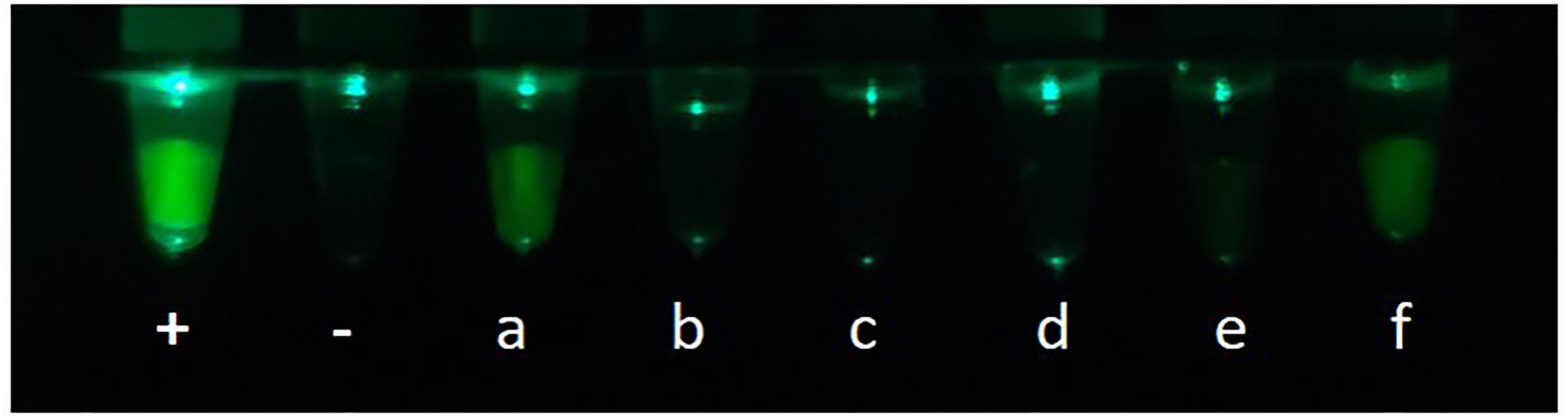
Partial visualization results of clinical samples using the 3D-printed visualization function cassette: +, Positive control; -, Negative control; (a–f) are clinical samples.

To assess the practical application of the CP-LAMP method, 61 DNA samples were used to test CP-LAMP. A total of 13 samples were detected as positive by traditional PCR methods, while 48 samples were negative. By comparison, 17 samples were detected as positive by the CP-LAMP method, 13 of which were detected as positive by both traditional PCR and CP-LAMP methods, but the other four samples were only detected as positive by the CP-LAMP method (**Table 4**). Thus, CP-LAMP achieved a superior detection rate compared with traditional PCR, and no positive samples were missed. Meanwhile, partial visualization results of clinical samples using the 3D-printed visualization function cassette are shown in **Figure 6**.

**Table 4:** Detection of suspected clinical ASFV samples.

Number of Samples	Traditional PCR		CP-LAMP		TaqMan probe real-time PCR	
	Positive	Negative	Positive	Negative	Positive	Negative
61	13	48	17	44	17	44

## Discussion

Since the first ASF outbreak in China in August 2018, the diseases has spread to almost 32 provinces, and huge numbers of pigs have been culled to halt further expansion, which has had a devastating impact on both pork production and food security (Li and Tian, 2018). Currently, neither an efficacious vaccine nor effective control strategies are available. Thus, a rapid, facile, and accurate on-site detection method is essential to help control the epidemic and minimize losses. In the present study, we established a CP-LAMP detection method for this purpose. The ASFV CP-LAMP method achieved fast, efficient, and specific amplification at a constant temperature (62°C) in a short time (within 40 min) using a DNA polymerase possessing high strand displacement activity (Yang et al., 2018). Furthermore, it achieved excellent detection performance without the need for advanced instrumentation or technological expertise (Mori and Notomi, 2020), hence it has the potential to be developed into a simple-to-use on-site molecular assay for diagnosis of ASFV in the field.

In the CP-LAMP ASFV detection method, based on the original primer sets, we targeted the 9GL gene sequence by designing a new fluorophore quencher-labeled cleaved probe with a ribonucleotide insertion; RNase H2 is only activated when the probe perfectly matches the mutant target, leading to the hydrolytic release of a quencher moiety, and consequently an amplified signal (Shen et al., 2020). Our CP-LAMP reaction can be measured in real time using a simple thermocycler to quantify fluorescence; it does not require any additional fluorescent intercalating dyes. Based on a 10-fold dilution series of positive plasmid solutions and their corresponding amplification curves, a standard curve equation was established. There was an excellent correlation between copy number and reaction duration, and the equation could be used for accurate quantification of unknown samples. A LAMP assay using EvaGreen as reported previously (Wang et al., 2020), but this method only achieved objective real-time detection, not quantitative detection. Another study reported an ASFV detection method that combined LAMP and image processing with the hue-saturation-value (HSV) color model. The colorimetric results of this LAMP assay can be used for semi-quantitative analysis of ASFV following HSV color space transformation (Yu et al., 2021).

Our CP-LAMP assay accurately detected ASFV without cross-reacting with other swine viruses, which demonstrates its high specificity. Furthermore, our CP-LAMP method only requires adding a fluorophore quencher-labeled probe, similar to the conventional LAMP method, and the other primer sets and dosage of reagents do not change. This makes is perfect for the highly sensitive standard LAMP strategy. Sensitivity analysis showed that the minimum detectable copy number was 13 copies/µL.

Compared with other methods, our method is more sensitive than traditional PCR assays for detection of ASFV DNA in field samples (Atuhaire et al., 2014). Moreover, the sensitivity of our CP-LAMP method is higher than that of the conventional LAMP assay (copy number = 330) (James et al., 2010), similar to a semi-quantitative colorimetric LAMP method (Yu et al., 2021) and a one-step visual LAMP assay using neutral red dye (Wang et al., 2021). Moreover, quantitative detection by CP-LAMP can be performed on an isothermal real-time instrument rather than a thermocycler, greatly decreasing costs for clinical use. In addition to real-time quantitative detection, we also used a home-made 3D-printed visualization function cassette for detection (**Figure 6**). Furthermore, a mobile phone can be used to both read the results and upload the data. Thus, our succinct operation process meets the needs of on-site diagnosis, and it may be applicable to other pathogens.

In summary, our method achieved real-time, quantitative, and sensitive detection of ASFV by replacing one primer with a probe without adding fluorescent intercalating dyes. The entire detection process can be completed under closed-tube conditions following a one-step sample addition process. Thus, our CP-LAMP detection platform achieves cost-effective, user-friendly, rapid, portable, and accurate POCT for ASFV.

## Data Availability Statement

The original contributions presented in the study are included in the article/supplementary material. Further inquiries can be directed to the corresponding authors.

## Ethics Statement

In this study, 61 DNA samples were provided by the Research Center for African Swine Fever Prevention and Control, South China Agricultural University, Guangzhou, China, which did not involve the isolation and identification of ASFV from samples of relevant sources. It did not include animal research and human research, and there were no ethical issues related to living animals and human. Ethics approval was not needed for this study from the Committee on the Ethics of Animal Experiments of South China Agricultural University, according to the Constitution on the Ethics of Animal Experiments of South China Agricultural University [Hua-nong-ban (2014)23hao], and the guidelines of our institution.

## Author Contributions

SW: Conceptualization, Methodology, Investigation, Visualization, Writing – original draft, Writing – review and editing. HS: Conceptualization, Writing – review and editing, Supervision. QL: Cassette design, Software. JH: Data curation, Formal analysis. CZ: Visualization, Resources. ZL: Data curation. MS: Data curation. JZ (8th author): Formal analysis. ML: Resources, Supervision, Project administration. YL: Validation, Writing – review & editing. JZ (11th author): Writing – review and editing, Supervision, Project.

## Funding

This work was supported by the Key-Area Research and Development Program of Guangdong Province (2019B020211005), the Special Topic on Emergency Prevention and Control of African Classical Swine Fever in Guangdong Province (2019B020211003), the Independent Research and Development Projects of Maoming Laboratory (2021ZZ003), the Special Fund for Scientific Innovation Strategy-construction of High Level Academy of Agriculture Science-Prominent Talents (R2020PY-JC001), and the Science and Technology Planning Project of Guangdong Province (2020A1515010950, 2021A1515011125).

## Conflict of Interest

The authors declare that the research was conducted in the absence of any commercial or financial relationships that could be construed as a potential conflict of interest.

## Publisher’s Note

All claims expressed in this article are solely those of the authors and do not necessarily represent those of their affiliated organizations, or those of the publisher, the editors and the reviewers. Any product that may be evaluated in this article, or claim that may be made by its manufacturer, is not guaranteed or endorsed by the publisher.
